# Transfer of Metals to the Aerosol Generated by an Electronic Cigarette: Influence of Number of Puffs and Power

**DOI:** 10.3390/ijerph19159334

**Published:** 2022-07-30

**Authors:** Brian Rastian, Chase Wilbur, Daniel B. Curtis

**Affiliations:** Department of Chemistry and Biochemistry, California State University, Fullerton, CA 92831, USA; brastian@csu.fullerton.edu (B.R.); cwilbur95@csu.fullerton.edu (C.W.)

**Keywords:** electronic cigarette, aerosol, metals, particulate matter, inhalation

## Abstract

Electronic cigarettes (e-cigarettes) are increasing in popularity despite uncertainties about their health hazards. Literature studies have shown that e-cigarettes may be a source of toxic heavy metal exposure to the user, but the mechanism by which metals are transferred from the e-cigarette parts into the aerosol plume that is inhaled by the user is poorly understood. The goal of this study was to quantify the potentially harmful heavy metals chromium, nickel, copper, and lead systematically during the simulated use of a mod-type e-cigarette in order to better understand the mechanism of metal transfer from the e-cigarette parts into the aerosol plume and into the liquid in the storage tank. Aerosol was collected and aliquots of the remaining liquid in the storage tank were collected from 0 to 40 puffs in 10 puff increments and analyzed with atomic absorption spectroscopy. It was found that the concentration of metals increased in both the aerosol and tank liquid the more times the e-cigarette was puffed, but at varying rates for each element and depending on the power applied to the heating coil. For copper, lead, and nickel, the concentrations of metals in the aerosol and tank increased with increasing power but for chromium, the concentration varied with power. Additionally, it was observed that chromium and nickel concentrations were greater in the aerosol than in tank liquid, consistent with the direct transfer of those metals to the aerosol from heating of the nichrome coil element used in this study. For copper and lead, the concentrations were similar or greater in the tank compared to the aerosol, consistent with transfer first into the storage tank liquid, followed by vaporization into the aerosol.

## 1. Introduction

Electronic cigarettes (e-cigarettes or ECs) are devices designed to deliver nicotine to the user by vaporization of a liquid and subsequent condensation into an aerosol of suspended particles. Although e-cigarette use is often touted as a safer alternative to tobacco combustion, the potential long-term health effects of e-cigarette use are not well understood [1,2,3,4]. The rapid increase of e-cigarette use, especially among adolescents, could present itself as an emerging public health concern [3,5,6,7,8,9], including potential associations with COVID-19 symptoms [10,11,12]. Although e-cigarettes are perceived to be safer than traditional combustion cigarettes, a specific health problem associated with e-cigarettes is the long-term exposure of users to potentially toxic metals.

Many metals and metalloids are essential for biological function, but chronic and acute exposure of humans to certain elements, such as nickel, chromium, lead, and arsenic, are known to have adverse toxicity when intake exceeds recommended amounts [13,14,15]. Humans are typically exposed to metals through a variety of pathways, including ingestion or inhalation of contaminated soil, dust, bioaccumulation in food, water, pesticides, and other sources [16,17,18,19,20,21,22]. Recent studies have shown that some of the metals found in electronic cigarettes accumulate in the central nervous system of mice after exposure, appear in various biomarkers of e-cigarette users, and have been shown to cause cellular damage [1,8,23,24,25,26,27,28,29,30,31,32,33,34,35,36].

The e-cigarette aerosol plume is produced by resistive heating of a metal coil that is in contact with a viscous solution (e-liquid) in a storage tank on the device. The e-liquid is composed of organic solvents (propylene glycol and glycerol) with varying amounts of nicotine and flavoring compounds. To produce an aerosol, the e-liquid is vaporized by “firing” the metal coil, applying a current to it, and heating it up to several hundred degrees, while the user inhales (“puffs”) through the mouthpiece [37,38]. The e-liquid vapor then condenses into an aerosol (a suspension of small particles in the air) at air temperature as the user draws air through the device. The user then inhales the aerosol plume, which delivers nicotine to the respiratory system, but also any other harmful compounds that are present [39]. The most widely used heating coil materials include Nichrome, an alloy made of nickel and chromium, and Kanthal, an alloy of iron, chromium, and aluminum [37,40], although technology continues to develop rapidly.

Metals have been measured in e-cigarette aerosol plumes [1,30,31,41,42], but the mechanism by which metals are transferred to the aerosol is not fully understood. A study by Aherrera et al. [34] tried to determine whether e-cigarette use is associated with increased nickel and chromium exposure, determined by non-invasive biomarkers, and found higher concentrations of nickel in the urine and saliva in participants who reported a shorter time to first use from waking, more frequent coil changes, higher urinary cotinine, and in users with higher nickel concentrations in the aerosol samples collected from their personal devices. For chromium, Aherrera et al. [34] found higher biomarker concentrations in users who reported consuming more e-liquid per week and concentrations in saliva increased drastically with higher chromium concentrations in the aerosol and tank samples from their personal devices. These findings indicate that e-cigarettes contribute to nickel and chromium exposure and that metals in the inhaled aerosol are a likely source of exposure. Studies have shown that the e-liquid itself is not typically a source of metals, indicating that firing the e-cigarette is necessary to transfer metals to the aerosol inhaled by the user [25,27,31,42,43]. In order to better understand the potential health impacts of e-cigarette use and to develop safer e-cigarettes, the mechanism by which metals are transferred from the e-cigarette components into the aerosol needs to be better understood.

Studies from the Talbot group [37,44,45] found that metal parts in the e-cigarettes are potential sources of metals to the aerosol. The source of the metalloid silicon was determined to be the sheath and wick, while solder was determined to be a potential source for tin and lead. Williams et al. [37] and Pappas et al. [46] determined that insoluble metal nanoparticles are potentially incorporated into the aerosol, possibly from mechanical breakdown of the heating filament or solder and that copper, tin, and lead could be coming from the copper wiring in the e-cigarette that is soldered to the heating coil [37,44,45]. Additional studies indicate similar findings, that the source of metals in the e-cigarette aerosol appears to be the metal heating coil and other parts, wiring, and solder within the e-cigarette itself [25,31,34,36,40,41,42,43,46,47,48,49,50,51].

Olmedo et al. [43] performed a study using 56 volunteer e-cigarette users in Maryland, USA, sampling the aerosol, tank, and dispenser liquids and analyzing for 15 elements. It was found that the concentrations varied by orders of magnitude, but on average that the concentrations of metals in the tank liquids were greater than the concentrations of metals in the aerosol and that the tank and aerosol concentrations were much greater than in the dispenser liquids prior to puffing. The Olmedo et al. [43] study illustrated an important correlation between metal concentrations in the storage tank e-liquid and in the aerosol. The findings support the hypothesis that metals are first transferred from the heating coil to the e-liquid in the tank and then from the e-liquid in the tank to the aerosol that is inhaled by the user. However, the focus of the Olmedo et al. [43] study was to obtain broad measurements from real-world users of tank-style e-cigarettes, with an array of different devices, voltages, coil types, e-liquids, and user conditions, such as how often the users changed the coil assemblies, which were acquired via a survey of the participants. The mechanism by which metals are dissolved into the e-liquid or incorporated into the aerosol was not systematically studied.

The purpose of the study presented here was to perform systematic, controlled measurements to better understand the mechanism by which heavy metals are transferred from the parts of an electronic cigarette into the aerosol that the user inhales and the potential variables that affect metal concentrations. An apparatus was developed to collect aerosol and tank liquid samples. A refillable mod-type (3rd-generation) electronic cigarette was chosen that allowed for control of the power and temperature settings on the coil and allowed the e-liquid to be tested separately from the e-cigarette. The e-cigarette in this study used a nichrome heating element, allowing for the potential transfer of nickel and chromium directly to the aerosol during heating, and therefore nickel and chromium were measured. The other elements studied, copper, and lead, were chosen due to their relatively high concentrations found in the studies cited above. Two experimental variables were studied here, the number of puffs taken and the power setting on the e-cigarette. It should be noted that the results of this study may only apply to the specific e-cigarette used here and that broader inference may not apply.

## 2. Materials and Methods

### 2.1. Electronic Cigarette

**Figure 1:**
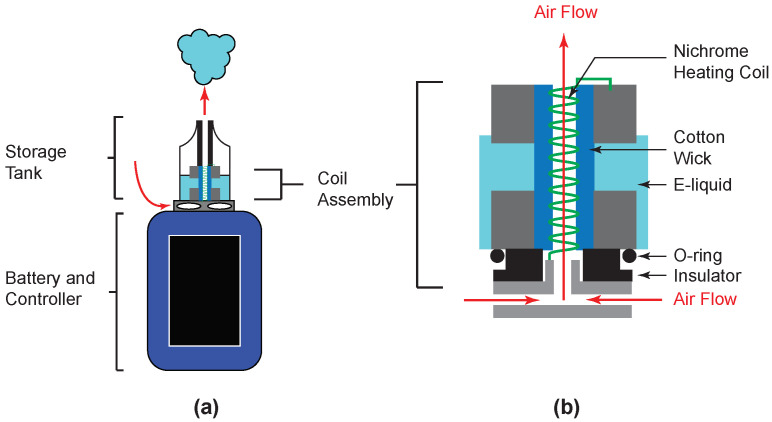
Schematic diagram of Vaporesso Revenger Mini E-cigarette used in this study. (**a**) General assembly with storage tank, coil assembly, and battery. (**b**) Magnified view of the coil assembly. For clarity, the assembly shown here has only one coil, while the GT8 coil assembly used in this study had four nichrome heating coils that heated simultaneously. Airflow through the device is indicated by red arrows.

Figure 1 shows a schematic diagram of the Vaporesso Revenger Mini used in this study and it will be used to illustrate how the aerosol is produced by an electronic cigarette. The GT8 coil assembly used in this study contained four separate nichrome heating coils in one assembly, each of which is fired simultaneously as the user presses a button on the battery/controller, although only one coil is shown in Figure 1 for clarity. The Vaporesso Revenger Mini fires when the button is pressed, not when airflow is detected as in some pod-type electronic cigarettes (e.g., JUUL). The Revenger Mini has air ports on the coil assembly that was maintained in the full open position for the work presented here.

The e-liquid in the Vaporesso Revenger Mini is in contact with a cotton wick through access port holes in the side of the assembly. The cotton wick draws liquid into contact with the heating coil. As a user inhales, the air flow passes into air vents and through the coil while the button is pressed and the heating coil, which makes electrical contact with the battery/controller, has current pass through it and heats to a temperature of several hundred degrees. The e-liquid is vaporized at the coil, then condenses in the air flow to generate the nicotine-containing aerosol that the user inhales.

Based on the manufacturer’s recommendation, the temperature profile used for the puffing experiments in this study was 400 ${}^{\circ }$F (204 ${}^{\circ }$C) which falls in the efficient range for vaporizing propylene glycol/glycerol solvent mixtures. This temperature allowed the solvent to vaporize and form aerosol particles. The e-liquid that was used throughout this project was commercially-purchased and is representative of typical e-liquids, and contained 70% glycerol and 30% propylene glycol by volume which would give an expected boiling point of 226 ${}^{\circ }$C for the mixture [52], slightly greater than the temperature used for this study.

### 2.2. Apparatus

**Figure 2:**
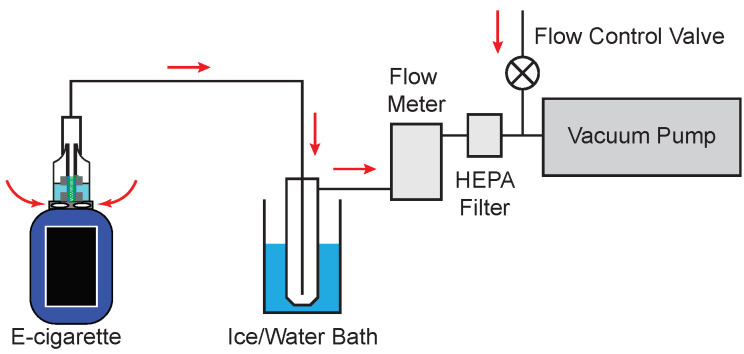
Experimental apparatus. Red arrows indicate direction of air flow through the system. A flow meter was used to measure the flow rate through the device, while a flow control needle valve was used to set the flow rate. A high-efficiency particulate air (HEPA) filter was placed in the line to remove excess particles before air flow entered the pump.

Figure 2 shows a schematic diagram of the apparatus used to collect the aerosol and tank samples. First, the Vaporesso Revenger Mini e-cigarette device filled with e-liquid was connected by a piece of Tygon tubing to the top of a glass impinger (Chemglass Life Sciences, Vineland, NJ, USA, CG-1820-01) submerged in an ice/water bath inside of a dewar. Then another piece of tubing was connected from the side of the impinger to a flow meter, followed by connections from the flow meter to a High Efficiency Particulate Air (HEPA) filter. Finally, tubing was connected from the HEPA filter to a mechanical vacuum pump. The direction of air flow is indicated by the arrows in Figure 2. A flow control needle valve was placed before the mechanical vacuum pump in order to control the flow rate by allowing room air to act as a make-up flow. Furthermore, a soap bubble-type flow meter (Sensidyne, St. Petersburg, FL, USA, Gilibrator 2) was placed before the HEPA filter in order to measure the flow rate prior to each experiment and was subsequently removed from the system before starting the puffing experiments. The HEPA filter was placed in front of the vacuum pump in order to prevent particles from entering the vacuum pump. The aerosol was collected in the impinger and the ice/water bath was used in order to facilitate re-condensation of the aerosol plume. Prior to aerosol collection, each impinger was cleaned with 1% nitric acid (Fisher Scientific, Pittsburgh, PA, USA, Trace Metal Grade) in ultrapure Nanopure water (Thermo Scientific, Pittsburgh, PA, USA, Nanopure Model D11971) with >18 MΩ·cm resistivity.

The electronic cigarette liquid (e-liquid) was purchased locally in 60 mL bottles and was Naked brand, Lava Flow (coconut, pineapple, and strawberry)-flavored with 6 mg/mL nicotine concentration. The choice of Lava Flow flavoring was due to the fact that it was the most popular flavor sold at the local retailer. Approximately 2 mL of e-liquid was placed in the storage tank and the e-cigarette was manually fired through multiple puffs. The flow rate of air through the e-cigarette was set at a constant 1110 ± 60 cm${}^{3}\;\min^{-1}$, similar to human breath rates, based on the Cooperation Centre for Scientific Research Relative to Tobacco, henceforth referred to as CORESTA [53] standard breath for electronic cigarette puffing. In slight contrast to the CORESTA standard, in this study the flow rate of air was set at a constant flow rate rather than mimicking the acceleration and deceleration present during an actual human breath.

An aliquot of e-liquid of known volume between 50 and 100 μL was extracted from the tank before puffing and after each subsequent 10 puffs and stored for analysis. Similarly, the aerosol was collected in a glass impinger submerged in an ice/water bath after each subsequent 10 puffs. This procedure was performed for a total of 40 puffs of the e-cigarette. After each 10-puff collection, the impinger was switched to a clean impinger for the subsequent 10 puffs. Thus, one impinger was used for puffs 1–10, a second impinger was used for puffs 11–20, a third for puffs 21–30, and a fourth for puffs 31–40. After 40 puffs, the e-cigarette tank was cleaned with Nanopure water and refilled and the procedure was repeated four to five times until enough liquid was collected, resulting in a total collected aerosol of approximately 500 μL of liquid volume for each impinger, 1–10, 11–20, 21–30, and 31–40. The overall collection efficiency of the aerosol was roughly estimated to be 25–40% based on the liquid volumes collected. One coil assembly was used for each collection, resulting in an individual coil assembly being used for 160–200 puffs. The coil assembly was then replaced with a new coil assembly and the entire process was repeated for a total of three independent measurements. Each puff was manually controlled, firing for 3 s with a rest period of 30 s between each firing (based on the standard CORESTA protocol No. 81).

Metal concentrations in the tank and aerosol samples were measured using a graphite furnace atomic absorption (GFAA) AAnalyst 600 spectrometer (Perkin Elmer, Waltham, MA, USA). Although ICP-MS is commonly used for metal analysis of e-liquids and aerosol [25,51,54], GFAA was chosen in this study for the ability to measure very small volumes of sample, and accessibility to the instrument, although fewer elements were able to be quantified compared to ICP-MS. Chromium, nickel, copper, and lead concentrations were measured by diluting the e-liquid samples by a factor of 2 with 1% ultrapure nitric acid (chromium, copper, and lead) and a factor of 10 for nickel. No further digestion of the samples were performed and dilutions were performed in 1% nitric acid [55,56]. Control experiments, described in the Appendix A, were performed and indicated that the room air was not a measurable source of any of the metals studied here.

## 3. Results

### 3.1. Influence of Number of Puffs

To better understand how metals are transferred into the aerosol, the concentration of chromium (Cr), nickel (Ni), copper (Cu), and lead (Pb) were measured in the captured aerosol and in aliquots of e-liquid removed from the storage tank of the e-cigarette as a function of the number of times the e-cigarette was puffed (and the coil fired). By comparing measurements of metal concentration in both the aerosol and the tank as a function of the number of puffs, this portion of the study provides insight into whether the metals enter the aerosol directly during the firing phase of the e-cigarette, or instead first dissolve into the e-liquid during the firing phase of the e-cigarette, followed by incorporation from the liquid into the aerosol.

In addition, measuring the concentrations of metals as a function of the number of puffs provides an important insight into whether or not the concentrations change as the coil is repeatedly fired or as the liquid volume decreases in the storage tank as the user puffs the e-cigarette. If so, users could be exposed to varying concentrations of metals as they puff on the e-cigarette. This study could potentially provide information to users to alter their use habits to reduce exposure to heavy metals while using e-cigarettes.

**Figure 3:**
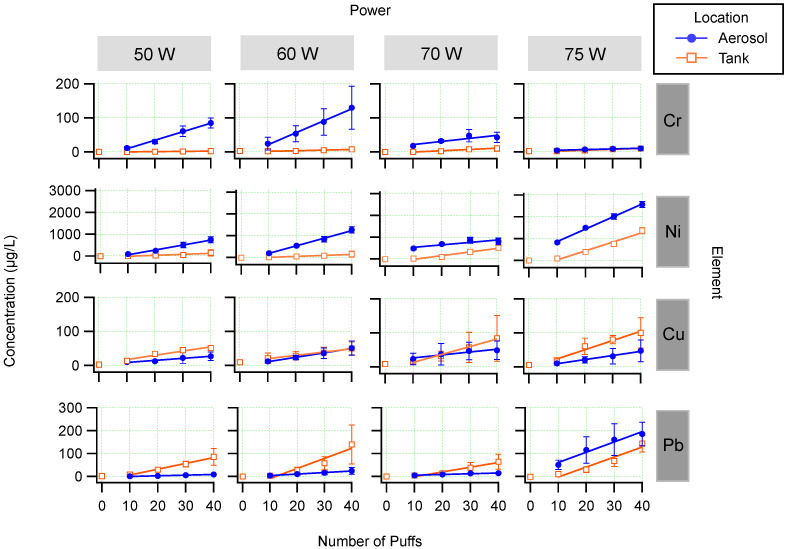
Results. Solid blue circles represent the concentrations in the collected aerosol and open orange squares represent the concentrations in the tank liquid. Note the varying vertical axes for different metals. Error bars are ± the standard deviations of three independent puffing experiments.

Figure 3 shows the concentrations of the metal ($\mu g\;L^{-1}$) in the collected aerosol and tank samples plotted as a function of the number of puffs. The columns represent different power settings and the rows represent the four elements studied here. Figure 3 shows that, on average, the concentration of these metals increases in the aerosol and in the tank as a function of number of puffs. The slope of the linear fit can be considered the average rate of increase in concentration per puff. The error bars in Figure 3 represent the standard deviations of the results of three independent puffing experiments for each power setting.

As explained in the experimental section, the points represent the incremental number of puffs. For example, the points shown at 10 puffs represent the aliquots collected from 1 to 10 puffs, the points shown at 20 puffs represent the aliquots collected from 11 to 20 puffs, etc. The tank measurements at zero puffs can be considered to be the e-liquid concentration from the dispenser after brief contact with the e-cigarette tank. The zero puff data was not included in the linear fits so that the rates of increase could be directly compared for the tank and aerosol measurements.

Table 1 lists the fit parameters for the linear fits shown in Figure 3. Using the slope of the linear fit, the rate of increase of the metal concentration can be determined as a function of the number of puffs. In all cases, the fits show a positive linear correlation between metalconcentration and number of puffs. In other words, the more the user puffs the e-cigarette without refilling the tank with fresh liquid, the higher the metal concentration to which they are exposed.

Figure 3 shows the concentration of chromium in the aerosol and tank plotted as a function of the number of puffs and various power settings across the top row. On average, the more times the e-cigarette was puffed (fired), the higher the concentration of measured chromium in the aerosol and the tank. The concentrations of chromium in the tank were relatively low and did not vary strongly as a function of the number of puffs. The slope of the linear fit can be considered the rate of increase in concentration per puff taken. The rate of increase was greater in the aerosol compared to the tank, except at a power of 75 W. There was significant variation in the rate of increase in the aerosol concentrations as the power varied (discussed in more detail below). The maximum rate of increase was at 60 W in the aerosol, with an average rate of increase of 3.5 ± 0.2 μg/L per puff (where the uncertainty is derived from the standard deviation of the fitted slope).

Figure 3 shows that there was a higher concentration of chromium present in the aerosol compared to the tank regardless of the power used, except when operating the device at 75 W. As the power was increased from 50 W to 60 W the concentration of chromium in the aerosol increased. Going from 60 W to 70 W the concentration of chromium in the aerosol decreased. Similarly, going from 70 W to 75 W the concentration of chromium also decreased in the aerosol. However, the concentration of chromium in the tank remained relatively constant and was relatively low even after 40 puffs of firing for all of the powers that were tested. Variations of concentration with power are discussed in more detail below.

The concentrations of nickel were the highest among the metals studied here. Figure 3 shows that, on average, the concentration of nickel increased as a function of the number of puffs, both in the aerosol and tank. This was true for all power settings studied. There was a higher concentration of nickel present in the aerosol than the tank regardless of the power used and the rate of increase was larger in the aerosol than the tank and varied with each power setting. The maximum rate of increase for nickel occurred for the aerosol at 75 W and was 58 ± 2 μg/L per puff.

Figure 3 shows the copper concentrations for the aerosol and tank as a function of number of puffs for the various power settings. On average, the concentration of copper increased in both the aerosol and tank as a function of number of puffs. Additionally, the concentration of copper in the tank and aerosol are similar for 50–70 W, and a higher concentration was observed in the tank liquid compared to the aerosol at a power setting of 75 W.

Figure 3 shows the lead concentration in the aerosol and tank as a function of number of puffs for each power studied. The concentration of lead in both the tank and aerosol increased on average with the number of puffs. As can be seen in Figure 3, there was a higher concentration of lead in the tank than in the aerosol at 50 W, 60 W, and 70 W, which is in contrast with chromium and nickel. The lead concentration remained relatively constant and low in the aerosol at 50 W, 60 W, and 70 W. However, there was a large increase in the lead concentration in the aerosol at 75 W. Variations in concentration with power are discussed in more detail below.

### 3.2. Influence of Power

For this study, the power applied to the coil was varied between 50 and 75 W, the high and low values recommended by the manufacturer for the GT8 coil-type used in this study. Importantly, the temperature of the coil was kept constant at 400 ${}^{\circ }$F (204 ${}^{\circ }$ C) regardless of the power applied. This removed the potential variable of the vapor pressure differences for each metal due to differing temperatures as a contributing factor to the heavy metal transfer to the aerosol during the vaporization phase.

**Figure 4:**
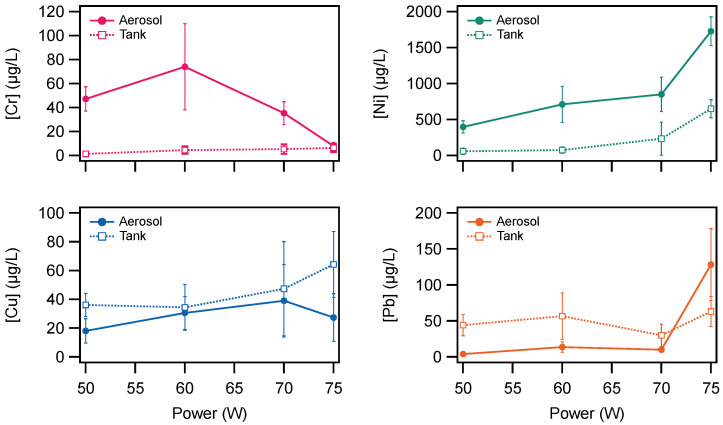
Mean concentration of metals in the aerosol (solid markers) and tank (open markers) from 10 to 40 puffs plotted as a function of power. Note: Error bars are ± the mean standard deviation of each concentration.

Figure 3 shows that there is a complicated relationship between the power and the concentrations of metals in the aerosol and tank liquids. To better describe this relationship, the measurements were averaged across the puffs, using the 10–40 puff values only (not the zero puff data). Figure 4 shows the mean concentration across all puffs plotted as a function of power.

The chromium concentration follows an unusual pattern compared to the other three metals. Figure 4 shows that chromium concentration was greater in the aerosol than the tank for all power settings, except for 75 W, where the concentrations were statistically similar. Additionally, it can be observed that the chromium concentration in the aerosol first increases between 50 W and 60 W, then decreases to 70 W, and finally decreases more at 75 W. The concentration in the tank liquid was low for chromium for all power settings and did not vary strongly with power.

Figure 4 shows that for nickel, the concentration was greater in the aerosol than the tank at all power settings. It can also be observed that the concentration of nickel in both the aerosol and the tank increased with increasing power. As noted above, the nickel concentration at each power setting was much greater than the other metals in this study.

Figure 4 indicates that, on average, the concentrations of copper and lead did not vary strongly with power. There were slight variations in the concentrations of copper and lead in the aerosol and tank, with a slight increase in the copper concentration in the tank with increasing power. However, it should be noted that the variations observed for copper were not statistically significant and do not exceed the reproducibility of the measurements.

In contrast to most of the chromium and all of the nickel measurements, the copper and lead measurements shown in Figure 4 show that the tank concentration was slightly greater than the aerosol concentration in all cases for the average of 10–40 puffs, except for a large increase in the observed lead concentration in the aerosol at 75 W. However, the values were similar across all power settings.

### 3.3. Aerosol/Tank Ratio

**Figure A1:**
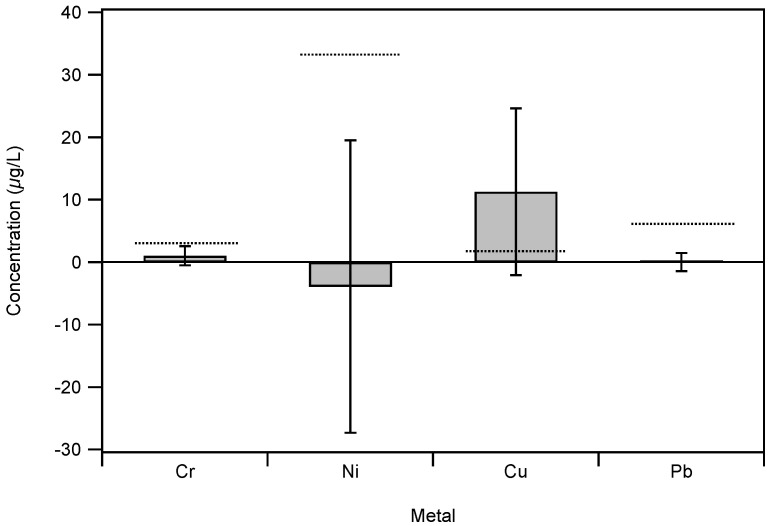
Mean of concentrations of chromium (Cr), nickel (Ni), copper (Cu), and lead (Pb) in the e−liquid placed in the tank measured before firing the e−cigarette. Error bars represent standard deviations of triplicate measurements. The dotted lines represent the Limits of Quantitation (LOQ) of the measurements.

The ratio of concentrations of metals in the aerosol or tank versus the concentration of metals in the e-liquid dispenser has been used to better understand how metals are transferred into the aerosol or tank liquid [43]. Therefore, the ratio of the metal concentrations in the aerosol to the concentrations in the tank liquid was used to give insight into the mechanism by which metals are transferred into the aerosol. As in other studies ([27,31,43,57]), the e-liquid from the dispenser used in this study had low metal concentrations, less than the limit of quantitation of the instrument for nickel, lead, and chromium (Figure A1), and therefore was not considered to be a source of metals in either the aerosol or tank liquids.

**Figure 5:**
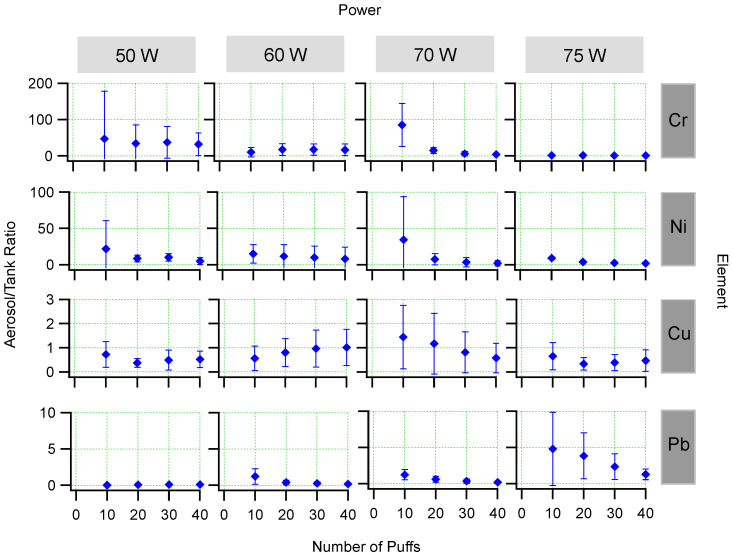
Aerosol/tank concentration ratio plotted for each element, power, and number of puffs. The error bars represent standard deviations of repeated puffing experiments.

**Figure 6:**
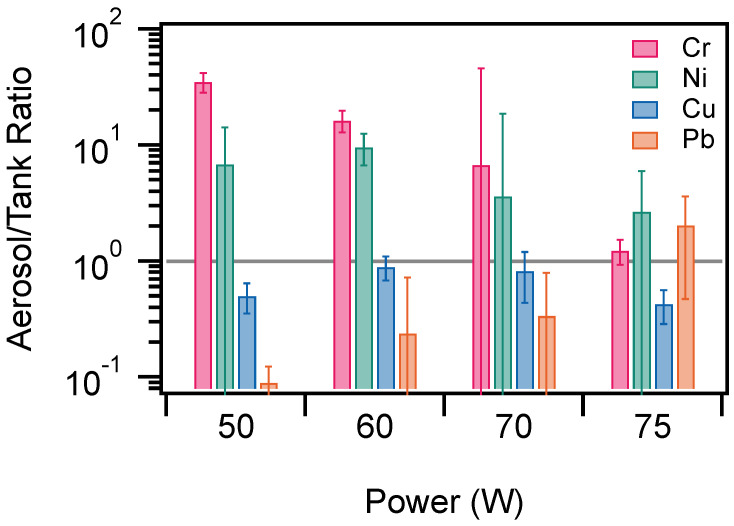
Mean aerosol/tank ratio plotted for each element and power. The gray horizontal line indicates an aerosol/tank ratio of one. The error bars represent the standard deviation of the mean aerosol/tank ratio over 10–40 puffs for each power.

Figure 5 shows the aerosol/tank concentration ratios for each element, power, and number of puffs. Figure 6 shows the mean aerosol/tank concentration ratio across all puffs for each element and power setting. In the cases of chromium and lead the aerosol/tank ratio is greater than one for all power settings. For copper, the aerosol/tank ratio is less than one in all cases. For lead, the aerosol/tank ratio was less than one for 50, 60, and 70 W, but was approximately two for 75 W.

Figure 6 shows the mean aerosol/tank ratio plotted for each element and each power setting. On average, the chromium and nickel aerosol/tank ratio is greater than one for each power and the ratio varies as a function of power, decreasing at higher power settings. Figure 6 shows that, on average, the aerosol/tank ratio is less than or near one for copper and lead. These results indicate that there is likely a different mechanism for the transfer of chromium and nickel to the aerosol as opposed to copper and lead. The chromium and nickel are greater in the aerosol, indicating that those metals are potentially transferred directly from the e-cigarette (likely from the heating filament) into the aerosol. In contrast, the copper and lead results indicate that the metals are transferred more efficiently into the tank liquid than the aerosol.

## 4. Discussion

The concentration of metals in the aerosol produced by the 3rd-generation mod-type electronic cigarette used in this study increased (on average) with increasing number of puffs. To reduce exposure to potentially toxic metals, we recommend that users change the coil assembly often and/or refill the tank often with fresh e-liquid (likely to have low metal concentrations). It should be noted that we can only state with certainty that the results of this study apply to the specific mod-type e-cigarette and specific coil type used here. Future studies should expand to other types of e-cigarettes and coil types.

The results are consistent with chromium and nickel becoming incorporated into the aerosol directly from the nichrome heating coil during the heating and puffing process. The results are also consistent with copper and lead becoming incorporated into the tank liquid during the heating and puffing process, followed by potential vaporization.

However, there is different behavior for chromium and nickel, despite both being found in the heating coil. The concentration of chromium in the aerosol varied with power, first increasing, then decreasing. For nickel, the concentration in the aerosol increased with increasing power. It would be expected that, if chromium and nickel were incorporated into the aerosol from mechanical breakdown of the heating element that they would both increase with increasing power However, chromium does not follow this trend. Although the observation that the chromium concentration first increased with increasing power, then decreased seems counterintuitive, this phenomenon has been observed in the literature before by Zhao et al. [31], for both chromium and manganese in an aerosol sample (tank measurements were not performed) for a variable-power open-device e-cigarette (SMOK) similar to the Vaporesso used in this study, but with a stainless steel sub-ohm resistance heating coil instead of the sub-ohm resistance nichrome coil material used in the Vaporesso. It is currently unclear why this phenomenon occurs, and future work will be needed to better understand it.

Even small power changes within the manufacturer’s recommended window make a difference in metal concentration in the aerosol. All of these experiments were conducted at the same temperature. Therefore, this must be due to the power only and is not a temperature effect. However, it is was not determined whether metals are transferred to the e-liquid through some sort of redox process or just through dissolution. It was determined that the e-liquid in the dispenser was not a major source of metals. Furthermore, leaving the coil in contact with the e-liquid without firing is not a major source of metals (see Appendix A).

Chromium and nickel are present in the coil itself while copper and lead are not. Chromium and nickel showed variations in the aerosol/tank concentration ratio that were different from the variations observed for copper and lead. This indicates that chromium and nickel are likely being transferred directly from the coil into the aerosol during vaporization of the e-liquid as the power is varied. This indicates that the location of the metals within the e-cigarette influences how the metals are transferred to the aerosol. Thus, it can be concluded that the coil itself plays an important role in the mechanism of metal transfer into the aerosol plume.

Overall, the results are consistent with the nichrome heating element being the source of chromium and nickel to the aerosol. The transfer of chromium and nickel takes place directly from the heating element into the aerosol, without intermediate transfer into the tank liquid. However, for copper and lead, the aerosol/tank ratio is less than one, indicating that the concentration in the tank is greater than the concentration in the collected aerosol. A ratio less than unity is consistent with transfer of copper and lead first into the tank liquid, then aerosolization of the tank liquid, with less than 100% efficiency of transfer of metals into the aerosol.

It is important to note that there was variation in the experiments, which we attribute to manually firing the e-cigarette and to small variations in the air flow rate between experiments, both of which may have affected metal concentrations. Additionally, Saleh et al. [58] noted that there may be variations between the coils used due to manufacturing differences. Regardless of these sources of variability, the average trends in the measurements were observable regardless of the dilution factor, method used to perform the measurements, and variation of volume of e-liquid in the tank and aerosol added from run to run. On average, the more times the e-cigarette is puffed, the higher the concentration of chromium, nickel, copper, and lead in both the tank and aerosol samples.

## 5. Conclusions

Potentially toxic metals have been observed in the aerosol and tank liquids in electronic cigarettes. However, the mechanism by which metals are transferred from the heating coil and other metal parts of the e-cigarette into the aerosol breathed by the user is not well understood. This study performed a systematic study to better understand the metal transfer process by measuring chromium, nickel, copper, and lead concentrations in the aerosol and tank liquid with varying number of puffs and power settings.

The results of this study indicate that the concentration of metals in the aerosol and tank liquid increase with an increase in the number of times the e-cigarette is puffed. On average, it was found that chromium and nickel concentrations were greater in the aerosol when compared to the tank liquid, while copper and lead concentrations were smaller in the aerosol when compared to the tank liquid. This result is consistent with the nichrome heating element being the source of nickel and chromium and that those elements are transferred directly to the aerosol during puffing.

It was observed that the power setting caused variation in the concentration of metals observed, depending on the element. Nickel concentration increased, on average, with increased power, while copper and lead did not vary significantly. As in previous studies, the concentration concentration of chromium in the aerosol was highest at an intermediate power setting and decreased when power was increased to higher power settings. Further investigations are needed to better understand the decrease in chromium concentration at higher power setting.

## Figures and Tables

**Table 1:** Fit parameters for linear fits of concentration vs. number of puffs. Slope (*m*) and intercept (*b*) are provided with standard deviations of the fits. The slope represents the average rate of increase in concentration per puff.

Element	Location	Power (W)	$m\pm s_{m}$ $\;(\mu {gL}^{-1}\;{\mathrm{puff}}^{-1})$	$b\pm s_{b}$ $\left(\mu {gL}^{-1}\right)$	R${}^{2}$
Cr	Aerosol	50	2.5±0.2	−16±4	0.9919
Cr	Aerosol	60	3.5±0.2	−14±5	0.9936
Cr	Aerosol	70	0.9±0.3	12±9	0.7830
Cr	Aerosol	75	0.20±0.03	3.0±0.8	0.9569
Cr	Tank	50	0.079±0.006	−0.6±0.2	0.9879
Cr	Tank	60	0.19±0.4	0±1	0.9345
Cr	Tank	70	0.37±0.05	−4±1	0.9640
Cr	Tank	75	0.26±0.01	−0.1±0.3	0.9957
Ni	Aerosol	50	22±2	−160±50	0.9881
Ni	Aerosol	60	35±2	−160±50	0.9951
Ni	Aerosol	70	11±4	420±120	0.7683
Ni	Aerosol	75	57±2	290±70	0.9966
Ni	Tank	50	5±1	−60±40	0.8535
Ni	Tank	60	5±1	−41±17	0.9651
Ni	Tank	70	17±2	−190±60	0.9709
Ni	Tank	75	42±5	−398±137	0.9721
Cu	Aerosol	50	0.60±0.09	3±2	0.9594
Cu	Aerosol	60	1.30±0.06	−2±2	0.9960
Cu	Aerosol	70	0.9±0.2	18±5	0.9119
Cu	Aerosol	75	1.2±0.1	−3±3	0.9870
Cu	Tank	50	1.2±0.2	5±6	0.9424
Cu	Tank	60	0.95±0.09	11±2	0.9824
Cu	Tank	70	2.3±0.2	−10±5	0.9863
Cu	Tank	75	2.7±0.4	−3±12	0.9520
Pb	Aerosol	50	0.28±0.03	−3.0±0.7	0.9828
Pb	Aerosol	60	0.67±0.06	−3±2	0.9867
Pb	Aerosol	70	0.33±0.06	2±2	0.9288
Pb	Aerosol	75	4.5±0.6	16±17	0.9612
Pb	Tank	50	2.5±0.2	−19±5	0.9901
Pb	Tank	60	4±1	−53±26	0.9122
Pb	Tank	70	2.1±0.3	−22±7	0.9663
Pb	Tank	75	4.4±0.9	−46±25	0.9206

**Figure A2:**
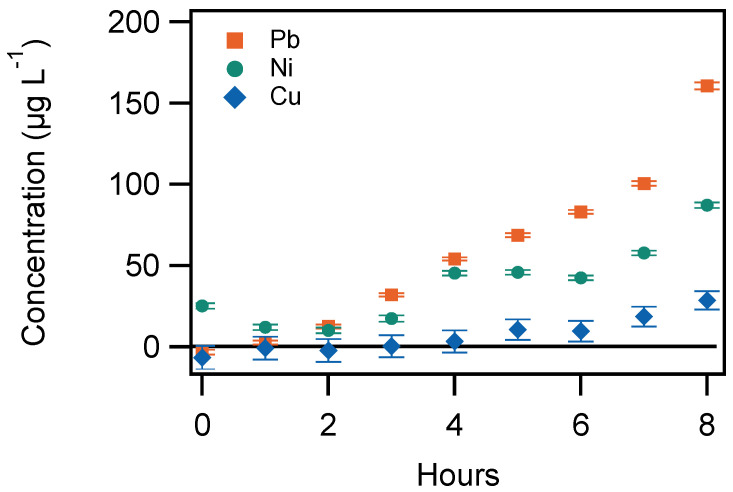
Concentrations of lead (Pb), nickel (Ni), and copper (Cu) for e-liquid over time with coil placed in e−liquid. Error bars represent standard deviations of triplicate measurements of individual liquid samples. Chromium concentration was less than the LOD in all cases and is not shown.

## Data Availability

The data presented in this study are openly available in figshare at https://doi.org/10.6084/m9.figshare.20288838.v1 accessed on 11 July 2022. (Under embargo while paper in review).

## Abbreviations

The following abbreviations are used in this manuscript:

Cr	Chromium
Ni	Nickel
Cu	Copper
Pb	Lead
GFAAS	Graphite Furnace Atomic Absorption Spectrometer
HEPA	High-Efficiency Particulate Air
LOD	Limit of Detection
LOQ	Limit of Quantitation

## Appendix A

### Appendix A.1. Instrument Parameters and Control Experiments

According to the manufacturer, the metal filament heating coils in the GT8 assembly were composed of nichrome wire. The composition was confirmed by removing, weighing, and dissolving one coil in heated aqua regia (1 M nitric acid: 3 M hydrochloric acid, Trace Metal Grade, Fisher Scientific). The digested sample was analyzed using flame atomic absorption spectroscopy (Buck Scientific, Model 210VSGP) and the metal coil composition was determined to be 83.22% nickel by mass, 3.35% chromium by mass, with the balance unknown [59]. This composition is consistent with the coil being composed of nichrome wire per the manufacturer and the balance element is likely iron.

The GFAA instrument parameters used in this study, e.g., wavelength, temperature program, graphite tubes, and lamp settings were based on the manufacturers recommendations. For copper and lead analysis on the GFAA instrument, matrix modifiers (Pd and Mg solution for copper, and NH${}_{4}$H${}_{2}$PO${}_{4}$ and Mg solution for lead) were used, per the manufacturer’s recommendation [59]. A multielement standard (100 mg/L, Perkin-Elmer) was diluted in 1% nitric acid (Trace Metal Grade, Fisher Scientific) and ultrapure water (Nanopure Model D11971, Thermo Scientific, 18 MΩ·cm resistivity) to produce sets of external standards, ranging from 0 to 100 μg/L.

A test experiment was performed to determine whether the room air in the lab had any chromium, copper, nickel, or lead present that could contaminate the e-cigarette aerosol during collection. A portable pump (Sensidyne, Gillian 5000) was used along with a Teflon filter (Pall Corporation) to collect the air sample and to test for metal analysis. Room sample filters and blank filters were extracted in 1% nitric acid and analyzed on the GFAA. No samples had nickel, chromium, copper, or lead concentrations greater than the limit of detection of the instrument. Thus, the room air in the lab, nitric acid solution, ultrapure water, and instrument sample cups were determined to not be a measurable source of heavy metals that are found in both the tank and aerosol samples in this study [59].

### Appendix A.2. Measurement Quality Control and Repeatability

The puffing experiments were performed in whole in triplicate, starting with a new coil each time. In addition, each GFAA measurement was performed in triplicate on the instrument. The variance between different experiments was greater than the variance for triplicate measurements, therefore instrument variation was not considered a large source of uncertainty in this study. However, variation was observed between experiments and this uncertainty is shown as error bars. Calibration standards and unknown samples were analyzed in triplicate and blank solution concentrations were subtracted. In all cases, the correlation coefficient of the linear calibration curves was $R^{2}>0.995$.

### Appendix A.3. Limits of Detection and Quantitation

Table A1 shows the limits of detection (LOD) for the elements analyzed in this study. Here we define LOD as three times the standard deviation of the response of the lowest concentration standard (measured in triplicate on the instrument) divided by the slope of the calibration curve, whereas the LOQ is ten times the standard deviation of the response of the lowest concentration standard (measured in triplicate on the instrument) divided by the slope of the calibration curve.

**Table A1:** Limits of detection (LOD) and quantitation (LOQ) of the elements analyzed in this study. Values are rounded to one significant figure.

Element	Instrument LOD ($\mu {g\;L}^{-1}$)	Instrument LOQ ($\mu {g\;L}^{-1}$)	Dilution Factor	Aerosol or Tank Sample LOD ($\mu {g\;L}^{-1}$)	Aerosol or Tank Sample LOQ ($\mu {g\;L}^{-1}$)
Cr	0.4	1	2	0.9	3
Ni	1	3	10	10	30
Cu	0.3	0.9	2	0.5	2
Pb	0.9	3	2	2	6

### Appendix A.4. Metal Concentrations in Unfired E-Liquid

In order to ensure that the metal concentrations observed during puffing were due to firing the coil and not simply due to contact with the metal parts in the e-cigarette tank and also to ensure that the metal concentrations in the e-liquid itself were not the source of metals, a control experiment was performed. For these measurements, the e-liquid was placed in the tank in contact with the coil assembly for a period of a few minutes, prior to beginning the puffing experiment, without firing the e-cigarette or flowing air through.

Figure A1 shows the concentration of each metal analyzed in this research project before firing the e-cigarette. As can be seen, there were relatively low concentrations of metals present in the e-liquid after being in contact with the metal coil for a few minutes. The dotted lines in Figure A1 represent the Limits of Quantification (LOQs) of the experiment, as shown in Table A1. It can be seen that for chromium, nickel, and lead, the concentrations are less than the LOQs, while for copper, the average concentration is greater than the LOQ, but still less than the values measured in the aerosol and tank. Therefore, it can be concluded that firing the e-cigarette contributes to the release of heavy metals in the tank and aerosol, and, similarly to other studies in the literature, that the concentration of these four metals was relatively low in the e-liquid itself, as purchased from the manufacturer. Therefore, the e-liquid itself is not a likely source for metals in the aerosol or tank.

It should be noted that the mean concentration of nickel was measured to be slightly less than zero. This values was less than the LOQ and should be considered to be zero within the statistical noise of the measurement rather than actually being a negative value.

In addition to the puffing experiments described above, a control experiment was performed where a coil was placed inside of a glass vial filled with e-liquid from the dispenser and the coil was allowed to soak and an aliquot was removed every hour (up to eight hours) to determine if having the coil assembly simply in contact with the e-liquid without firing might be a significant source of chromium, nickel, copper, and lead. Figure A2 shows the concentrations of copper, lead, nickel, and chromium as a function of contact time without firing the coil. Each puffing experiment in this study took less than one hour to complete and concentrations were higher than the metal concentrations in the control. Additionally, a long-term measurement showed that the concentration of chromium was less than the limit of detection of the graphite furnace atomic absorption spectrometer for a GT8 coil assembly stored in the e-liquid for 43 days, indicating the chromium does not readily dissolve in the e-liquid without firing the coil. Therefore, chromium is not shown in Figure A2.

The non-puffing contact-only experiments indicate that the concentrations of the chromium, copper, nickel, and lead that were present in the tank samples from the puffing experiments were due to firing and not due to the coil simply being in contact with the e-liquid over the typical time period of the puffing experiments. Therefore, simple contact with the e-liquid in the storage tank is not considered to be a major source of metal transfer to the aerosol in this study. However, it should be noted that the concentrations of metals did increase over time, indicating that users who fill an e-cigarette with e-liquid and store the device may be exposed to higher concentrations of metals than users who fill and immediately use the e-cigarette.
